# PLA_2_R binds to the annexin A2-S100A10 complex in human podocytes

**DOI:** 10.1038/s41598-017-07028-8

**Published:** 2017-07-31

**Authors:** Maryline Fresquet, Thomas A. Jowitt, Edward A. McKenzie, Matthew D. Ball, Michael J. Randles, Rachel Lennon, Paul E. Brenchley

**Affiliations:** 1 Wellcome Centre for Cell-Matrix Research, University of Manchester, Manchester, UK; 2 Division of Cardiovascular Science, University of Manchester, Manchester, UK; 3 Division of Cell-matrix Biology & Regenerative Medicine, University of Manchester, Manchester, UK; 4 Manchester Institute of Biotechnology, University of Manchester, Manchester, UK; 5 Central Manchester University Hospitals NHS Foundation Trust, Manchester, UK

## Abstract

Phospholipase A_2_ receptor (PLA_2_R) is a member of the mannose receptor family found in podocytes in human kidney. PLA_2_R is the target of the autoimmune disease, membranous nephropathy, characterised by production of anti-PLA_2_R autoantibodies which bind to the podocyte. However the function of PLA_2_R in health and in disease remains unclear. To gain insight into the molecular mechanisms of PLA_2_R function, we searched for its endogenous binding partners. Proteomic analysis identified annexinA2 as a potential interactor with the extracellular domains of PLA_2_R. We confirmed that PLA_2_R binds to annexinA2-S100A10 (A2t) complex with specific high affinity to the S100A10 component. The binding occured within the PLA_2_R NC3 fragment and was increased in acidic pH. Furthermore Ca^2+^ promoted the association of the PLA_2_R-A2t complex with phospholipid membranes *in vitro*. Within the podocyte, all three proteins were enriched in the plasma membrane and organelle membrane compartments. PLA_2_R co-localised with S100A10 at the cell surface and in extracellular vesicles. This novel interaction between PLA_2_R and the A2t complex offers insights into the role of PLA_2_R in podocytes and how autoantibodies might disrupt PLA_2_R function. The ability of podocytes to secrete vesicles containing PLA_2_R provides a route for engagement of PLA_2_R with the immune system.

## Introduction

Phospholipase A_2_ receptor (PLA_2_R) is a member of the mannose receptor family consisting of Endo180, mannose receptor (MR) and DEC-205^1^. These receptors share a common domain organisation including an N-terminal Cys-rich domain, a fibronectin type II domain, a variable number of C-type lectin domains (CTLDs), a single transmembrane region and a short cytoplasmic tail. The MR family receptors exist in at least two conformations, an extended linear or a bent conformation, depending on the environmental pH^2^. MR, Endo180, DEC-205 and PLA_2_R have been shown to function as endocytic receptors in various cell types, sharing the classic cytoplasmic NP(X)Y motif which is thought to control recycling from the endosomal system back to the plasma membrane^3^. PLA_2_R, like other family members, is rapidly internalised from the cell membrane via clathrin-coated pits into the endosomal system^4^. The lack of classical phosphorylation sites in the cytoplasmic tail implies that these receptors may signal through a cytoplasmic binding partner. It has been shown that the PLA_2_R cytoplasmic NPYY motif specifically interacts with the PTB domain of the dok2 adaptor protein^5^.

MR and Endo180, but not PLA_2_R and DEC-205, bind and internalise collagen isoforms^6^ although other studies suggest PLA_2_R may interact with collagen in other ways^7, 8^. Early studies implicate group IB and IIA secreted phospholipase A_2_ as a natural ligand for the mouse PLA_2_R receptor but this has not been confirmed as yet for the human counterpart^9^.

PLA_2_R is the least studied member of the MR family. In humans, PLA_2_R has a restricted distribution with variable low to medium levels within podocytes in the glomerulus^10, 11^ whereas in mice, PLA_2_R is absent from podocytes but is present in the lung and immune tissues^12^. In humans, PLA_2_R has been shown to be the target of an autoimmune disease, membranous nephropathy, a rare glomerulopathy, where anti-PLA_2_R antibodies deposit in the glomerulus affecting podocyte function and inducing nephrotic range proteinuria^10^. Lack of expression in rodent kidney has negated the development of *in vivo* experimental models of anti-PLA_2_R pathophysiology. It is imperative, therefore, to understand the role of PLA_2_R in human podocytes in order to understand why and how PLA_2_R engages with the immune system to generate autoantibodies. This knowledge may reveal how these anti-PLA_2_R antibodies affect podocyte function to cause disease. Human podocyte cell culture is an established model for investigating podocyte cell biology^13^ and we have used this system to investigate the functional role of PLA_2_R.

We have previously modelled the structure of the human recombinant PLA_2_R and confirmed that it exhibits a pH-dependent conformational switch as shown by other members of the MR family^14^. However, autoantibody binding is independent of the pH conformational change whose function remains to be explained. In this study, we describe the expression of PLA_2_R in human podocytes in culture. We identify a co-binding partner for the extracellular sequence of PLA_2_R in podocytes in pull-down experiments followed by proteomic analysis. We show that the PLA_2_R interaction with the main binding partner is pH-dependent and occurs within NC3, an N-terminal fragment of PLA_2_R which exhibits at least two epitopes recognised by autoantibodies. We describe this novel PLA_2_R complex on the podocyte plasma membrane and in secreted extracellular vesicles. These findings provide new insights into the biology of PLA_2_R in podocytes and suggest a novel mechanism of PLA_2_R presentation to the immune system.

## Results

### PLA_2_R in podocytes and its binding partners

**Figure 1:**
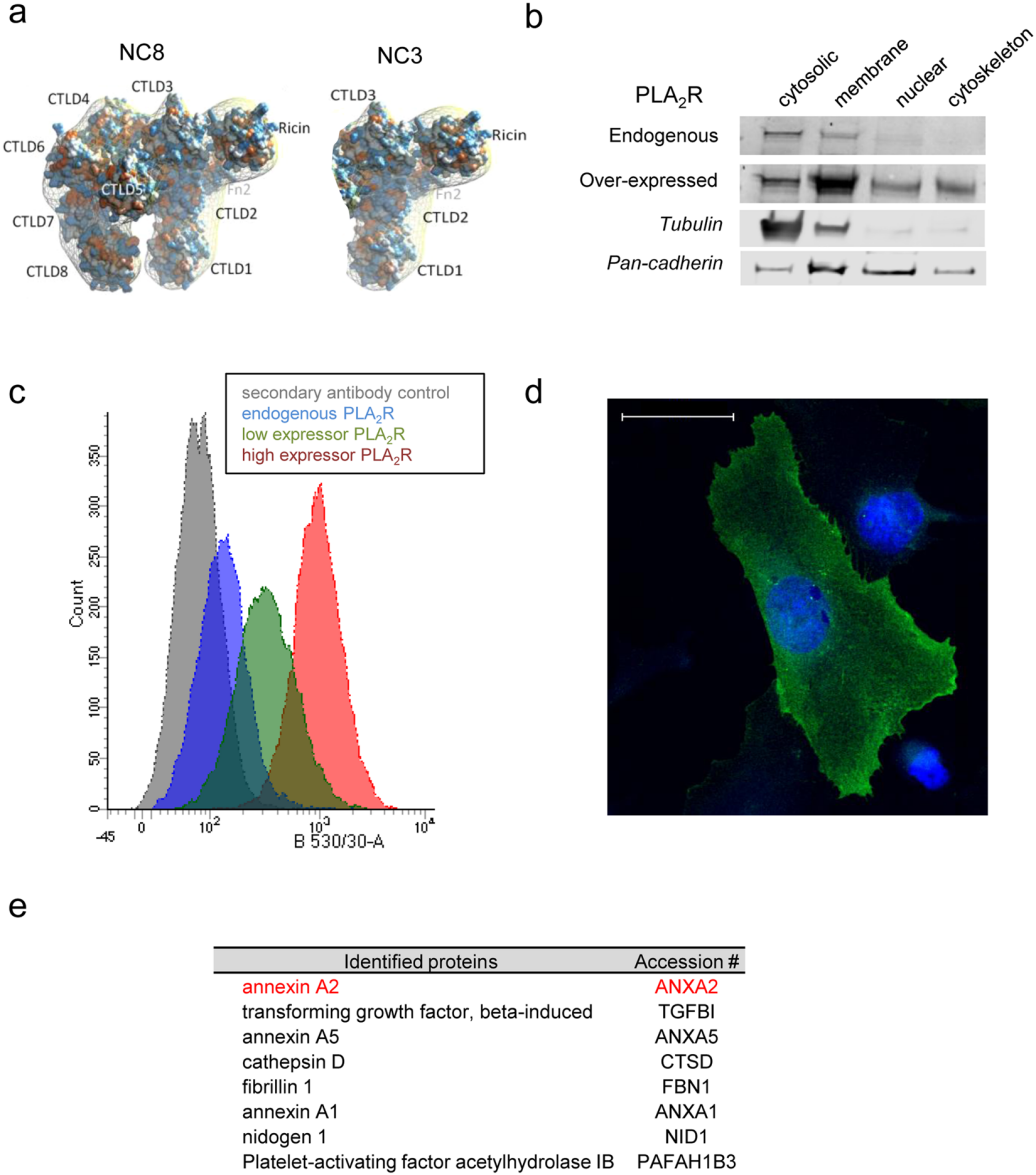
PLA_2_R in podocytes and its binding partners. (**a**) Structural models of the extracellular sub-domains of a long fragment PLA_2_R (NC8) comprising the N-terminal CysR domain (or ricin domain), fibronectin type II domain and 8 C-type lectin domains (CTLD), and a shorter fragment ending after the first 3 C-type lectin domains (NC3). (**b**) Western blot analysis of fractionated cellular protein from wild type and over-expressing PLA_2_R podocytes. Podocytes proteins were fractionated using the subcellular protein extraction kit. Each extracts were analysed by western blotting using antibodies against PLA_2_R, tubulin and cadherin, markers of cytoplasmic and membrane proteins, respectively (full length blots are presented in Supplementary Fig. 1). The results show an enrichment of PLA_2_R protein in the membrane and membrane organelles fraction. (**c**) Overlay flow cytometry histograms of wild type (dark blue line) and over-expressing PLA_2_R podocytes (low expressor, green line; high expressor, red line) stained with mouse anti-PLA_2_R 12-6-5. Negative controls were cells labelled with mouse IgG (grey line). (**d**) Immunofluorescence image of PLA_2_R over-expressing podocytes revealing cell surface expression of the PLA_2_R receptor using mouse anti-PLA_2_R (under 40x lens). Scale bar, 50 µm. (**e**) List of extracellular matrix (ECM) proteins bound to the extracellular domains of PLA_2_R identified by mass spectrometry.

PLA_2_R is characterised by a modular structure made up of extracellular domains, a transmembrane domain and a short cytoplasmic tail. We previously expressed the extracellular domains of PLA_2_R, referred as full length NC8 and a shorter fragment, NC3^14^. 3D models of these recombinant proteins are illustrated in Fig. 1a. Western blotting analysis of lysates extracted from differentiated wild type podocytes revealed a low level of endogenous PLA_2_R partitioned between cytosolic and membrane extracts (Fig. 1b) prompting us to generate an over-expressing cell line for selected experiments. We chose to transfect podocytes with the shorter fragment of PLA_2_R (NC3 plus transmembrane and cytoplasmic tail) as over-expression of full length PLA_2_R has been shown to induce senescence^15^. Podocytes stably transfected with the NC3 construct showed significantly higher levels of expression in all extracts with subcellular fractionation identifying an enrichment of the PLA_2_R protein at the cell membrane (Fig. 1b). Flow cytometry analysis of podocytes labelled with anti-PLA_2_R validated the increased expression of the PLA_2_R receptor in the transfected cells compared to wild type cells and confirmed its localisation at the cell membrane (Fig. 1c). Immunofluorescence imaging of over-expressing podocytes using a mouse monoclonal anti-PLA_2_R (NC3 specific, Supplementary Fig. 2) confirmed that PLA_2_R is present on the plasma membrane in cultured cells (Fig. 1d).

To gain further insight into the molecular mechanisms of PLA_2_R function at the plasma membrane, we searched for binding partners of PLA_2_R. They were isolated from wild type human podocytes using two methodologies and then analysed by mass spectrometry (MS). 484 proteins were identified across all samples, 50 were extracellular matrix proteins (when compared with the Matrisome database, http://matrisomeproject.mit.edu) and 32 proteins bound to PLA_2_R NC8 (Supplementary Table 1). Eight proteins were detected by both methods and we selected annexinA2, the top hit based on detection in all three experiments and highest spectral count (Fig. 1e). We chose two of the lesser candidates fibrillin-1 and nidogen-1 and confirmed no significant binding to PLA_2_R by SPR (data not shown).

### Defining the receptor complex

**Figure 2:**
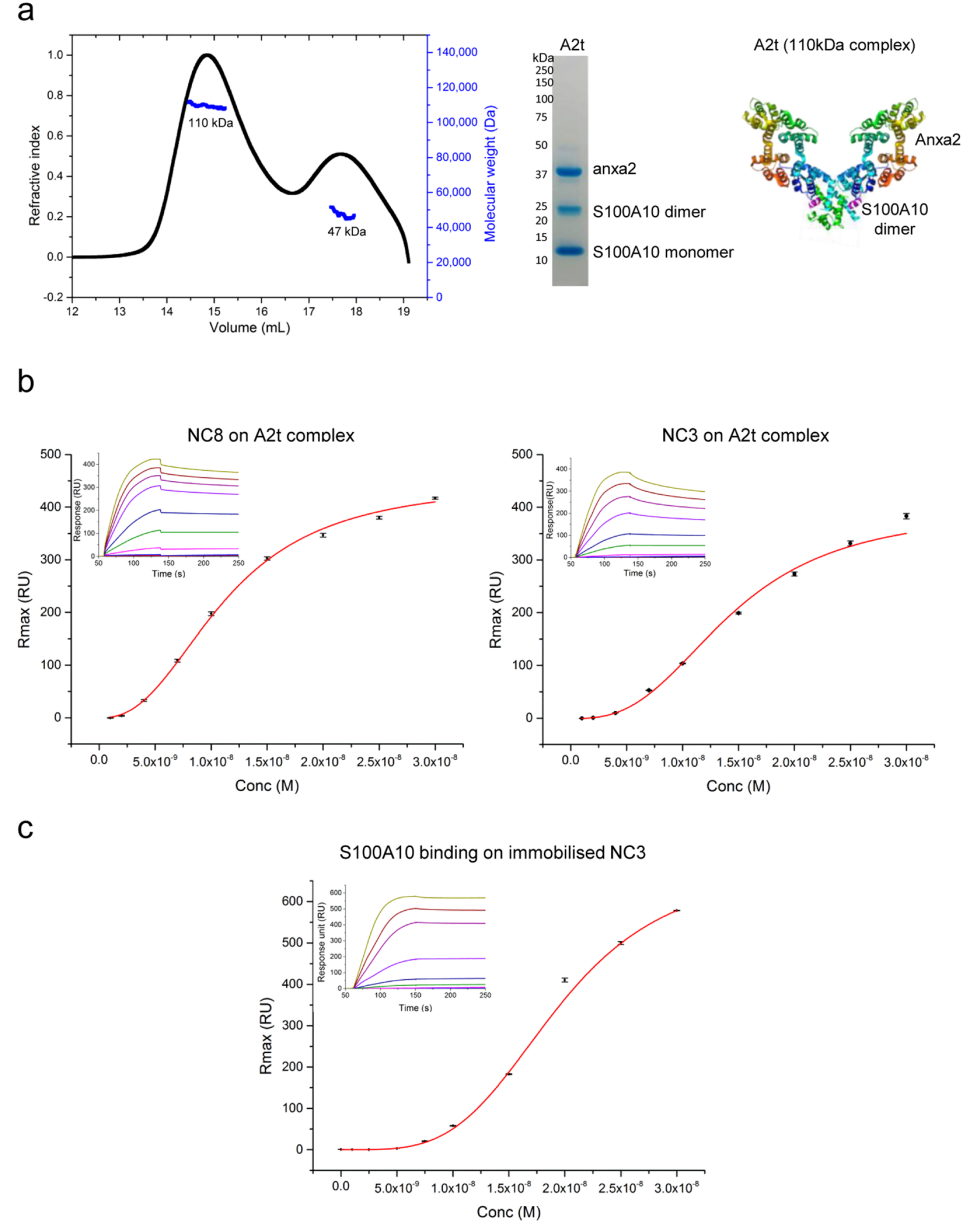
PLA_2_R interacts with A2t complex via S100A10. (**a**) *Left*, characterisation of Annexin2-S100A10 complex in solution using Multi Angle Laser Light Scattering (MALLS). The elution profile reveals 2 peaks, 110 kDa referred as A2t (Anxa2/S100A10 heterotetramer) and 47 kDa complex. *Middle*, 10 µg of purified complex ran on a non-reduced SDS-PAGE gel and stained with Instant Blue confirm the presence of both Anxa2 and S100A10 proteins in various oligomeric forms. *Right*, schematic representation of A2t complex made of a S100A10 dimer (10.2210/pdb1BT6/pdb) and two Anxa2 molecules (10.2210/pdb2hyw/pdb). (**b**) SPR data showing direct interaction with PLA_2_R fragments and A2t complex. Purified PLA_2_R NC8 and NC3 at various concentrations (0 to 300 nM) were injected over the surfaces of immobilised A2t. The maximal response (RU_max_) were obtained from the sensograms (inset) and plotted against the increasing concentrations of injected PLA_2_R. Each data point in both experiments is the mean of 3 repeats. The equilibrium dissociation constant, *K*_*D*_, was calculated by fitting the data to the Hill equation using nonlinear regression. (**c**) Representative sensorgrams derived from injections of different concentrations of purified recombinant S100A10 protein over immobilised PLA_2_R NC3. Results were obtained after reference subtraction.

MS analysis identified annexinA2 as a possible interactor across all three experiments (Supplementary Table 1). AnnexinA2 is a multi-functional protein whose subcellular localisation and functions are tightly regulated by its post-translational modifications. AnnexinA2 is mainly found in the cytoplasm and is lacking a signal sequence for secretion^16^. However when annexinA2 and S100A10 interact to form a heterotetrameric complex (A2t) of an S100A10 dimer with two annexinA2 molecules (Fig. 2a, schematic), it can translocate from the cytoplasm to the extracellular plasma membrane^17^.

We generated the recombinant A2t protein complex in bacteria using a dual expression vector followed by affinity purification. The complex was then separated by size exclusion chromatography and characterised in solution using Multi Angle Laser Light Scattering (MALLS) and SDS-PAGE analysis. The elution profile revealed 2 peaks, 110 kDa referred as A2t (Anxa2/S100A10 heterotetramer) and 47 kDa complex (Fig. 2a). Only the fraction containing the fully characterised heterotetramer of the correct size (110 kDa) and composition was selected for further studies.

We tested the binding between PLA_2_R and recombinant A2t complex by surface plasmon resonance (SPR) and found a very high affinity interaction with positive cooperativity with both NC8 and NC3 fragments of PLA_2_R (Fig. 2b). On and off-rate analysis using standard 1:1 Langmuir model was not possible due to the cooperative nature and tightness of the fit (very low dissociation). Fitting of the data using Hill model provided information on the kinetics of both weaker and stronger aspects of the interaction with a K_D_ of 11.3 nM and 14.8 nM for NC8 and NC3 respectively. The Hill coefficients were 2.4 for NC8 and 2.8 for NC3 which suggests multiple binding sites and/or conformational rearrangement upon binding. We then analysed annexinA2 (data not shown) and S100A10 individually and showed that PLA_2_R NC3 interacts very tightly with S100A10 (K_D_ = 19.3 nM, Fig. 2c). We attribute the lack of S100A10 detection in our MS results to the low abundance and low molecular weight of this particular protein. Small proteins (S100A10 is 11 kDa) generate very few tryptic peptides which sometimes are difficult to detect in a complex mixture.

These data demonstrate that the binding site with the heterodimeric complex is located in the N-terminal region of PLA_2_R where the major autoimmune epitope in the CysR domain (Cysteine Rich also known as ricin-like domain) is also located. This is the first evidence that PLA_2_R is another receptor interacting with the A2t complex.

### Does PLA_2_R bind A2t complex at the plasma membrane?

**Figure 3:**
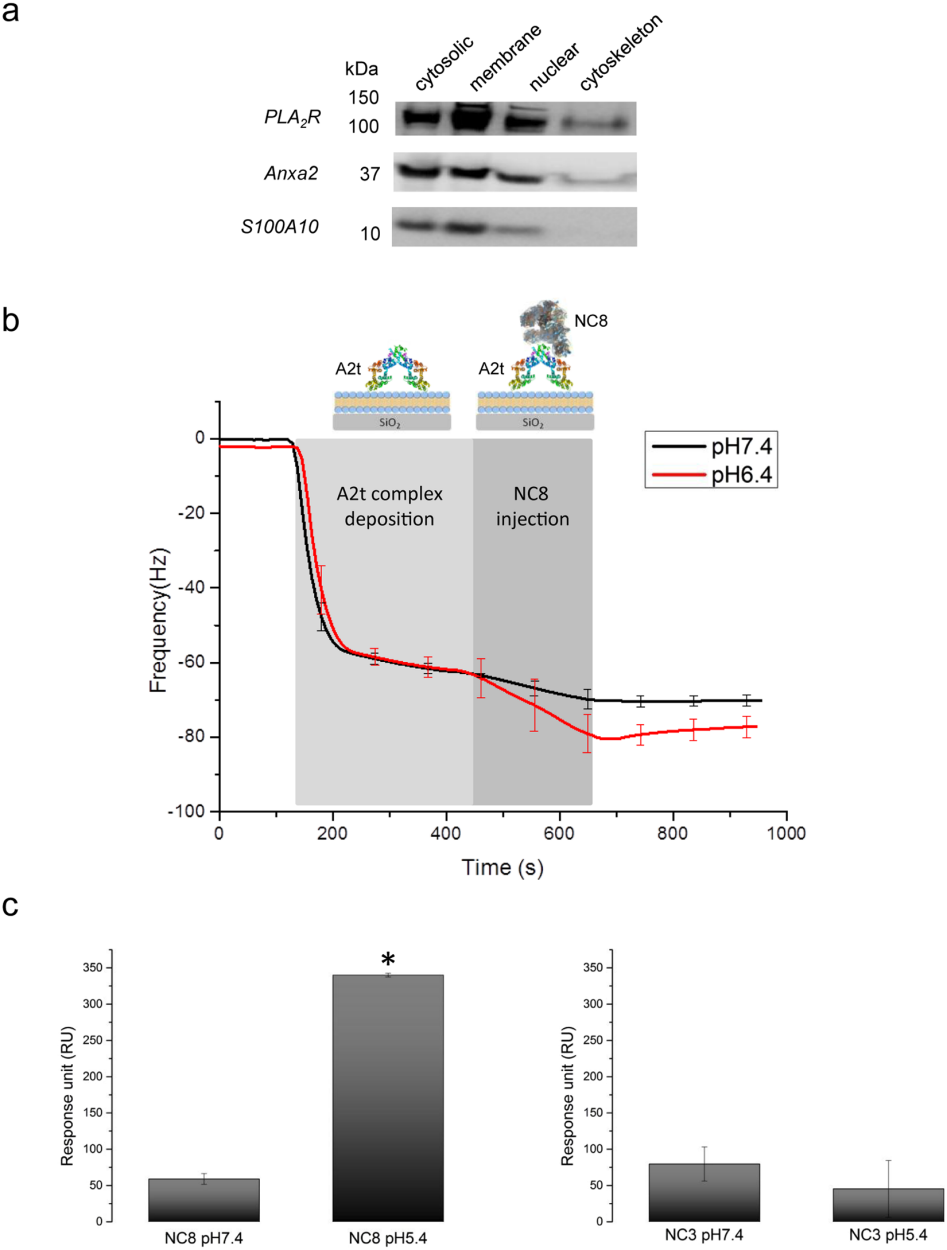
PLA_2_R binding to A2t complex is increased in a pH-dependent manner. (**a**) Western blot analysis of fractionated cellular protein from over-expressing PLA_2_R podocytes. Podocytes proteins were fractionated using the subcellular protein extraction kit. Each extracts were analysed by western blotting using antibodies against PLA_2_R, Anxa2 and S100A10 (full length blots are presented in Supplementary Fig. 3). The results show an enrichment of all three blotted proteins in the membrane and membrane organelles fraction. (**b**) The complex formation between PLA_2_R and A2t at different pHs was determined in real time using quartz crystal microbalance with dissipation (QCM-D). A2t complex was injected onto a preformed lipid bilayer. A2t protein bound to the lipids and formed a stable layer. PLA_2_R NC8 incubated either in buffer pH 6.4 or pH 7.4 was then added while monitoring the frequency change. An increase in bound PLA_2_R NC8 to A2t complex was detected in acidic pH 6.4 compared to neutral pH 7.4. (**c**) Measurements of the binding level of PLA_2_R NC8 and NC3 to S100A10 at different pHs using SPR. A maximum binding was obtained between PLA_2_R NC8 and S100A10 at pH 5.4 confirming the QCM-D data. The different pHs did not significantly affect the binding of NC3 to S100A10. Statistical significance was evaluated using ANOVA test. *P < 0.05.

We sought to define the subcellular location of A2t and PLA_2_R in podocytes. Fractionation of a podocyte extract identified an enrichment of all three proteins PLA_2_R, S100A10 and annexinA2 in the plasma membrane and membrane organelles fractions (Fig. 3a, Supplementary Fig. 3).

A2t complex is known to be associated with cell membranes of a specific lipid composition^18^. We therefore investigated whether PLA_2_R could still associate with the A2t complex while linked to the cell membrane. We further characterised the interaction of PLA_2_R and A2t on DOPC lipid bilayers with or without 10% DOPS lipids using Quartz Crystal Microbalance with Dissipation (QCM-D). We were able to measure mass changes occurring at the surface and found a specific calcium-dependent interaction of the A2t complex as previously demonstrated^18^ (Supplementary Fig. 4). Importantly, PLA_2_R NC8 and NC3 were also able to bind the membrane bound A2t complex in a non Ca^2+^-dependent manner, presumably through the available S100A10 molecules. Moreover the binding of PLA_2_R NC8 to A2t complex was increased at a pH less than 6.4 compared to neutral pH (Fig. 3b). The binding level of PLA_2_R NC8 and NC3 to S100A10 at varying pHs was also measured using SPR (Fig. 3c). A maximum binding was obtained between PLA_2_R NC8 and S100A10 at pH 5.4 confirming the QCM-D data. The binding of NC3 to S100A10 was not significantly affected over this range of pH.

### PLA_2_R in vesicles

**Figure 4:**
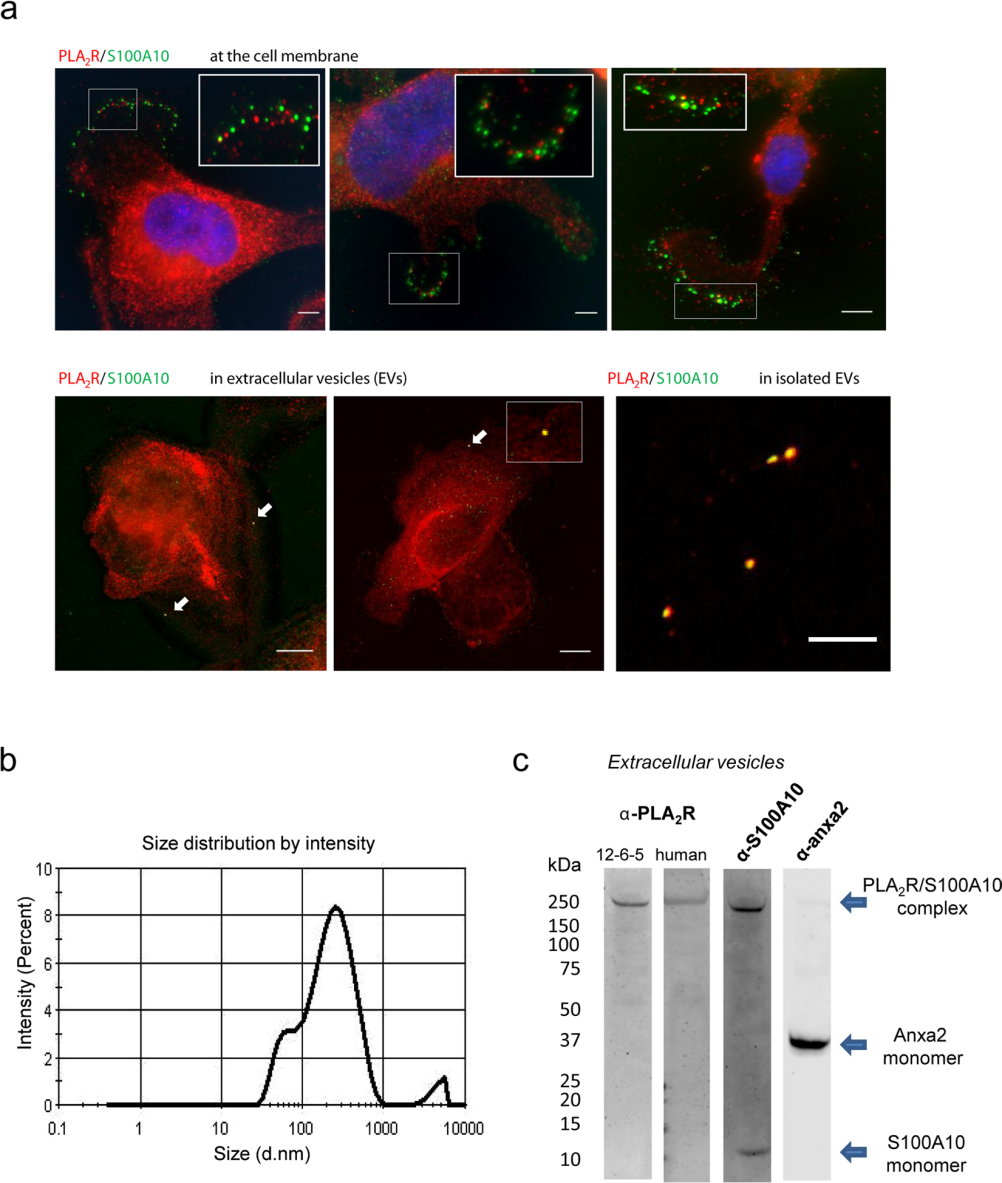
PLA_2_R is found in vesicles secreted by podocytes. (**a**) Co-localisation of PLA_2_R/S100A10 at the cell surface and in vesicles. Podocytes cultured on coverslips were fixed and co-stained using antibodies against S100A10 (green) and PLA_2_R (red) mAb 12-6-5. Merged images demonstrate some regions of overlap (yellow) between the PLA_2_R and S100A10 at the cell membrane (*top panel*) and in vesicles (*left bottom panels*). Scale bars, 10 μm. Purified extracellular vesicles (EVs) secreted from podocytes were incubated on poly-D-lysine coated glass slide and co-stained for PLA_2_R and S100A10 showing positive staining for both proteins. (**b**) Representative size distribution by intensity profile of extracellular vesicles (EVs) measured by Dynamic Light Scattering (DLS). EVs intensity distributions reveal two characteristic peaks occurring at ~60 nm and ~200 nm. These two values are in agreement with the average values reported in literature for exosomes and microvesicles, respectively. (**c**) Vesicles characterisation. Isolated EVs from serum-free conditioned cell culture media of differentiated podocytes were analysed by western blotting using anti-PLA_2_R, anti-S100A10 and anti-Anxa2 antibodies. A ~250 kDa band was detected by both anti-PLA_2_R and anti-S100A10 antibodies corresponding to the tightly associated PLA_2_R/S100A10 complex. Anxa2 was also detected in the EVs fraction as a monomer around ~37 kDa suggesting it has dissociated from the complex under denatured conditions.

Immunostaining of differentiated podocytes showed some co-localisation of PLA_2_R/S100A10 at the cell surface and within extracellular vesicles (Fig. 4a). At the edge of the cell, a punctate pattern of staining for PLA_2_R and S100A10 was observed (*top panel*). Both proteins were found in the vicinity of each other with some overlap in places. Significant staining of PLA_2_R and S100A10 was detected within the cell but no co-localisation between the two proteins (Supplementary Fig. 5a). To investigate whether these vesicles might be components of an active secretion pathway, we isolated an extracellular vesicles (EVs) fraction after overnight culture by ultracentrifugation. EVs were harvested and precipitated from serum-free conditioned cell culture media of differentiated podocytes. Purified EVs were incubated on poly-D-lysine coated glass slide and co-stained for PLA_2_R and S100A10. The vesicles showed positive staining for both proteins (*bottom panel*).The size distribution of the purified vesicles was determined by Dynamic Light Scattering (DLS). Two populations of vesicles were distinguished, a group of vesicles with a diameter between 60–80 nm and another group comprising of bigger vesicles measuring around 200 nm (Fig. 4b). The enriched vesicles fraction was then analysed by western blotting using anti-PLA_2_R, anti-S100A10 and anti-Anxa2 antibodies. A ~250 kDa band was detected by both anti-PLA_2_R and anti-S100A10 antibodies corresponding to the tightly associated PLA_2_R/S100A10 complex (Fig. 4c). Anxa2 was also detected in the EVs fraction as a monomer around ~37 kDa suggesting it had dissociated from the complex under the denaturing conditions.

## Discussion

In this study we have identified and characterised a novel molecular interaction between PLA_2_R and the A2t complex. We first sought evidence for the nature and form of PLA_2_R in human podocytes. Endogenous PLA_2_R could be detected at the cell surface of cultured podocytes by both western blotting and flow cytometry. Transfection of PLA_2_R (NC3) transmembrane receptor increased expression levels significantly in the membrane fraction. We next investigated the possible role of PLA_2_R as part of a larger receptor complex on the cell surface or in extracellular matrix. Interrogation of podocyte protein extracts for binding to PLA_2_R using MS analysis identified a number of annexin candidates but only annexinA2 was a consistent binding partner across three experiments. Although annexinA2 can exist alone it is more often complexed with a smaller classic binding partner, S100A10^19^. AnnexinA2 binding to S100A10 occurs through the N-terminal 14 aa^20, 21^. However, we did not see a signal for S100A10 in these experiments. In order to test and confirm the interaction with annexinA2, we expressed annexinA2 as the heterotetrameric complex (A2t) comprising two Annexin A2 molecules combined with an S100A10 dimer which has shown to be the common receptor supporting a wide range of functions in diverse cell types^22^. We used SPR to show that both PLA_2_R fragments (NC8 and NC3) could bind to the 110 kDa A2t complex and that the binding was accounted for by the S100A10 component. This implies that S100A10 was present but undetected in our initial pull-down experiments. It is known that some small proteins can be difficult to detect in MS so we suspect this to be a possible cause for its absence during our analysis.

The A2t complex bridges phospholipid vesicles, binds cell membranes, organises F-actin in lipid rafts and shows dynamic remodelling of the actin cytoskeleton. It is intimately involved in epithelial tight junction assembly and receptor trafficking. Intracellularly, the two partners in the complex exhibit different roles; S100A10 binds and trafficks plasma membrane proteins such as the 5-HT1B receptor^23^, TRPV5 and TRPV6^24^ and TASK-1^25^ whereas the Anxa2 components of A2t serve to anchor the complex in a Ca^2+^-dependent manner to the plasma membrane. The translocation of the A2t complex is known to be caused by changes in intracellular pH^26^. On the cell surface, the A2t platform interacts with tPA and plasminogen controlling the conversion to plasmin on endothelium^27^, and binds AHNAK to act as a target for dysferlin in repairing injured epithelial cells^28^.

Our study identifies PLA_2_R as a receptor that uses the A2t platform in podocytes. PLA_2_R specifically binds to the S100A10 component of A2t using a site in the NC3 fragment of PLA_2_R, containing both the major autoantibody epitope in the CysR domain, and an epitope in CTLD1^29^. Whether autoantibody binding to PLA_2_R can disrupt the interaction with S100A10 is currently under investigation. If podocytes use A2t as other epithelial cells do, this then implies that PLA_2_R may be at the heart of actin cytoskeleton reorganisation and tight junction assembly, two functions known to be modulated early in proteinuric membranous nephropathy.

The short cytoplasmic tail of PLA_2_R and its obvious lack of classic phosphorylation sites led to the proposal in cancer biology studies that cell membrane localised PLA_2_R might bind to a coreceptor that signals through JAK2^30^. Certainly in Anxa2, the key amino residues Tyr^23^, Ser^11^ and Ser^25^ are targets for phosphorylation by Src family tyrosine kinases and serine kinases^31, 32^. The interaction of the cytoplasmic motif NPYY in PLA_2_R with dok2 adaptor protein also offers additional signalling potential as dok2 is known to interact with 120 rasGAP, a potent inhibitor of Ras signalling. Whether the PLA_2_R-A2t complex can mediate signals from PLA_2_R crosslinking by antibody through these pathways needs investigation.

We investigated the distribution of PLA_2_R, annexinA2 and S100A10 throughout the podocyte by western blotting of cell extracts enriched for different cell compartments. All three proteins were present and enriched in the plasma membrane/organelle membrane fraction. We confirmed the binding of PLA_2_R-A2t complex in a calcium dependent manner to phospholipid bilayers.

We previously demonstrated that NC8, but not NC3, fragments of PLA_2_R undergo a conformational change at pH less than 6.2 which was unrelated to autoantibody binding^14^. We now demonstrate that this conformational change at acidic pH enables NC8 to bind more readily to A2t. We have also observed that the binding is strongly cooperative suggesting conformational rearrangement of either the A2t or PLA_2_R. This effect may promote the capture of PLA_2_R by the A2t complex within vesicles for trafficking to the cell membrane. As these vesicles fuse with the plasma membrane at a neutral pH^33^, they would deliver the PLA_2_R-A2t complex to the cell membrane and allow PLA_2_R to dissociate from the complex. We confirmed the *in vitro* co-localisation of PLA_2_R with S100A10 at the cell surface and in extracellular vesicles from podocytes by immunofluorescence staining. Both pH dependent effects on the conformation of PLA_2_R and on the binding of PLA_2_R to S100A10 may have significant biological meaning for endocytic and secretory vesicle function in podocytes in health and in cases of membranous nephropathy.

Further analysis of the EVs preparation isolated from podocyte conditioned culture medium validated the presence of PLA_2_R, Anxa2 and S100A10. As yet, the precise nature of these vesicles is unknown but are CD9 positive, a known vesicle marker (Supplementary Fig. 5b). Podocytes have been demonstrated to display a range of vesicles including endocytic vesicles, trafficking vesicles to plasma membrane and exosomes for secretion^34^. *In vivo*, urinary vesicles originating from podocytes have been isolated and characterised in normal urine and urine from patients with nephrotic syndrome including two patients with membranous nephropathy^35^. In this study the authors detect PLA_2_R in samples from nephrotic patients but not in healthy controls. However other studies described urinary vesicles isolated from healthy individuals to be containing PLA_2_R, Anxa2 and S100A10^36, 37^ This implies that podocyte vesicle secretion may be quantitatively altered in disease. If podocytes secrete vesicles containing PLA_2_R into the nephron *in vivo*, this provides a novel mechanism for immune cells downstream of the glomerulus to phagocytose, process and present PLA_2_R peptides for antibody production. Whether this mechanism is dysfunctional in membranous nephropathy and contributes to initiation of the autoimmune disease can now be tested.

## Methods

### Cells

Immortalised human podocytes^13^ (a kind gift of Dr MA Saleem) expressing endogenous PLA_2_R were cultured in RPMI media supplemented with 10% FCS and ITS. Proliferative podocytes were thermo shifted to 37 °C and typically differentiated for 5 days.

To generate the PLA_2_R NC3 over-expressing podocyte cell line, a construct containing the N-terminal portion of PLA_2_R (Methionine1 - Proline663) fused with the transmembrane region and the cytoplasmic tail (Proline1390 - Glutamine1463) was first engineered and cloned into a lentiviral pCDH vector (SystemBio). Viral particles were obtained by transfection of 293 T with psPAX2 and pMD2.G (Addgene) and pCDH-NC3. Conditioned media containing viruses was collected after 4 days and filtered through a 0.45 µM filter. Immortalised human podocytes at 60% confluency were transduced with the virus in infection media (RPMI containing polybrene, 1:1000 dilution) for 48 hrs and infected cells selected using puromycin (2.5 µg/ml).

### Production of mouse monoclonal anti-PLA_2_R (NC3)

Monoclonal anti-PLA_2_R (clone 12-6-5, raised against NC3 fragment) was produced in mouse using a standard immunisation protocol (Proteogenix SAS). Hybridoma cell lines were generated and the resulting supernatant was affinity purified using protein G column. Our mouse anti-PLA_2_R (12-6-5) was compared with a rabbit anti-PLA_2_R peptide antiserum (Sigma Aldrich, Poole, UK) by immunostaining on normal kidney tissue and membranous nephropathy biopsy tissue (Supplementary Fig. 2). The monoclonal anti-NC3 antibody was also used for western blotting and immunofluorescence as shown in Fig. 4. Anti-PLA_2_R (12-6-5) is available from Abcam and Novus Biologicals, UK.

### Subcellular fractionation of podocyte extracts

The subcellular fractionation of a confluent 10 cm^2^ plate podocyte extracts was performed using the ProteoExtract®Subcellular Proteome Extraction Kit (S-PEK, *Merck Chemicals Ltd*) following the manufacturer’s instructions. The kit enables the differential extraction of proteins according to their subcellular localisation and yields proteins in their native state. ProteoExtract®Subcellular Proteome Extraction Kit yields the total proteome fractionated into four subproteomes of decreased complexity. With Extraction Buffer I cytosolic proteins are released (fraction 1). Subsequently, membranes and membrane organelles are solubilised with Extraction Buffer II, without impairing the integrity of nucleus and cytoskeleton (fraction 2). Next, nucleic associated proteins are enriched with Extraction Buffer III (fraction 3). Components of the cytoskeleton are finally solubilised with Extraction Buffer IV (fraction 4).

### Protein expression and purification

The codon-optimised clone of human extracellular PLA_2_R (NC8) was modified to generate the smaller PLA_2_R fragments NC3 expression vector as previously described^14^. The resulting constructs were transfected into HEK 293-EBNA1 cells (human embryonic kidney cells; Invitrogen) using Lipofectamine 2000 reagent (Invitrogen) and the secreted proteins were purified using nickel affinity chromatography (GE Healthcare).

Bacterial codon optimised genes encoding Flag-tagged AnnexinA2 and His-tagged S100A10 were synthesised (Genscript). Both genes were sub-cloned into pETDuet-1 vector (Novagen) for co-expression of both target genes. Constructs were freshly transformed into competent JM109 DE3 *E.coli* cells (NEB). Cells were grown to an OD600 of 0.6, chilled on ice and 0.2 mM IPTG added and cells left shaking overnight (220 rpm) at 18 °C. Cells were harvested (5000 rpm at 4 °C) and lysed in chilled 0.3 M NaCl, 25 mM Tris-HCl pH 8.0, 1% Triton X-100, protease inhibitor cocktail minus EDTA (Sigma) buffer. Resuspended cells were sonicated 7 × 20 second bursts at 20% setting with cooling in between and then clarified by centrifugation at 17,000 rpm at 4 °C. Clarified lysate was then purified by nickel affinity chromatography (Qiagen) using an imidazole gradient (10–50 mM) and then eluted in 250 mM Imidazole buffer. Protein was de-salted in 150 mM NaCl, 25 mM Tris-HCl pH 7.9 buffer using a PD10 column (Generon). Protein was further purified by passing over a HiLoad 16/60 Superdex 200 PG column run in the same buffer. Protein was concentrated using vivaspin 3 kDa MWCO spin filters (Generon).

### Mass spectrometry workflow

Three separate experiments were designed in order to determine the binding partners of the extracellular domains of PLA_2_R. One MS sample originated from a pull down experiment whereby 40 µg of purified PLA_2_R NC8 were bound to Dynabeads His-Tag (Life Technologies AS, Norway) and subsequently incubated with soluble matrix proteins secreted from podocytes (serum-free media) for 20 minutes. After stringent washes, the complex was eluted with 100 µl His-elution buffer (300 mM imidazole, 50 mM sodium phosphate pH 8, 300 mM NaCl, 0.01% Tween-20). Non-coated beads were also incubated with soluble matrix proteins and used as control (for non-specific interactors). This sample is referred as “Pull down NC8 coated beads” in Supplementary Table 1. The other two samples were prepared using QCM-D. For “QCM-D NC8” sample, a solution of 15 µg/ml of purified PLA_2_R NC8 (in 10 mM BisTris pH7.4 + 1 mM Mg^2+^/Ca^2+^, flow rate = 20 µl/min) was injected on a silicon dioxide crystal until saturation. Soluble matrix proteins were then flown through the immobilised NC8, and the adsorbed mass was monitored. After washing with running buffer, the stable complex was eluted from the crystal using SDS-loading buffer. For the “QCM-D lipid bilayer/NC8” sample, the SiO_2_ chip was first coated with a lipid bilayer containing streptavidin. Biotinylated PLA_2_R NC8 was then bound to the surface allowing a controlled orientation of the protein. The incubation of the matrix and elution of the complex were performed as described for sample 2. All three samples were run on a SDS-PAGE gel for 3 min at 200 V, stained with Instant Blue for 15 min, and destained with MilliQ water overnight. Protein bands (gel top) were excised and identity confirmed by in-gel trypsin digests MS analysis.

### Characterisation of Anxa2-S100A10 (A2t) complex pre-SPR

The purified A2t complex was further purified and characterised using Multi Angle Laser Light Scattering (MALLS). 500 µl (=500 µg) of purified A2t protein was injected on a Superdex 200 column (GE Healthcare). Light scattering intensity and eluant refractive index were analysed using ASTRA version 6 software to give a weight-averaged molecular mass (Mw). The composition of the eluted fractions were confirmed by western blotting.

### Surface Plasmon Resonance (SPR)

The binding of recombinant A2t and S100A10 to PLA_2_R was investigated using a Biacore T200 instrument using a CM5 sensor chip. In the first instance, the Biacore was used to investigate the binding affinity between PLA_2_R fragments NC8 and NC3 and immobilised A2t complex. Purified recombinant A2t was immobilised in sodium acetate pH 4.5 at 25 °C onto two different flow cells of the CM5 sensor chip and resulted in 300 RU bound level. All subsequent binding experiments were performed in 10 mM BisTris pH 7.4, 150 mM NaCl, 0.05% Tween-20. PLA_2_R fragments were injected at concentrations ranging from 0 to 300 nM at a flow rate of 30 µl/min. Samples were injected for 2 min, dissociated for 10 min, regenerated with two successive 5 s injections of 10 mM NaOH and then stabilised for 20 min before the next injection. The analyte was simultaneously passed over a blank flow cell, and this baseline was subtracted from the experimental flow cell. After subtraction of each response value from the control cell, the maximal response (RU_max_) were obtained from the sensograms and plotted against the increasing concentrations of injected PLA_2_R. Each data point is the mean of 3 repeats. The equilibrium dissociation constant, *K*_*D*_, was calculated by fitting the data to the Hill equation using nonlinear regression (OriginPro v.9).

Kinetic runs were also performed between S100A10 and immobilised PLA_2_R NC3 using BisTris running buffer. The analytes were injected for 150 s at 30 µl/min at concentrations ranging from 0 to 200 nM.

### Quartz crystal microbalance with Dissipation (QCM-D)

The complex formation between PLA_2_R and A2t was determined in real time using quartz crystal microbalance with dissipation (QCM-D). QCM-D simultaneously monitors changes in resonance frequency (∆*f*) and dissipation (∆*D*) in real time. ∆*f* primarily measures changes in the mass attached to the oscillating sensor surface (a silicon dioxide crystal in this case), while ∆*D* measures properties related to the viscoelasticity of the adsorbed layer. QCM-D measurements were performed on a Q-sense E1 with standard flow module.

Prior to lipid bilayer adsorption, the crystal was equilibrated with 20 mM HEPES, 150 mM NaCl, 2 mM CaCl_2_ to establish a baseline. 1 mg/ml DOPC (1,2-dioleoyl-sn-glycero-3-phosphocholine) or DOPC/10% PS (1,2-dioleoyl-sn-glycero-3-phospho-L-serine) (Avanti Polar Lipids Inc) were prepared by extrusion from chloroform stocks. Liposome vesicles were diluted 1:10 into HEPES buffer and were flowed at 25 µl/min for 10 min. Once the lipid bilayer was formed, purified A2t complex was injected at a flow rate of 50 µl/min until saturation. NC8 and NC3 at pH 7.4 or pH 6.4 were flowed over the preformed complex. Frequency and dissipation shifts after a 3 min exposure of the bilayer to each protein were recorded using Q-soft and analysis of layers was performed using the Qtools software (Q-sense®, Gothenburg, Sweden).

### Microscopy - Immunofluorescence

Images from Fig. 4a (*top panel*) were collected on an Olympus BX51 upright microscope using a 40×/0.5 UPlan FLN objective and captured using a Coolsnap ES2 camera (Photometrics) through Metavue v7.8.4.0 software (Molecular Devices). Specific band pass filter sets for DAPI, FITC and Texas red were used to prevent bleed through from one channel to the next. Images from Fig. 4a (*bottom panel*), and Supplementary Fig. 5a were collected on a Leica SP8 inverted confocal microscope using a *40x* and *100x* oil objectives. The confocal settings were as follows; pinhole *1 airy unit*, scan speed *4000 Hz unidirectional*, format *2048 *×* 2048*. Cross-talk between channels was eliminated by ensuring minimal spectral overlap by imaging single stained controls with both scan sequences. Images were processed and analysed using Fiji/ImageJ software (version 1.46r; National Institutes of Health, Bethesda, MD, USA).

### Extracellular Vesicles (EVs) isolation

Differentiated immortalised human podocytes expressing endogenous PLA_2_R were cultured in three 10 cm^2^ plates. EVs were harvested from 24 ml of serum-free conditioned cell culture media and centrifuged at 300 × *g* for 10 minutes to remove detached cells. Supernatant was collected and filtered through 0.22 µm filters (Merck Millipore) to remove contaminating apoptotic bodies and cell debris. Clarified cell culture media was then centrifuged in a Beckman Coulter Optima™ L-90K ultracentrifuge at 100,000 × *g* at 10 °C for 2 hours with a SW40 Ti rotor to pellet vesicles. The supernatant was carefully removed, and crude vesicles-containing pellets were resuspended in ice-cold PBS. A second round of ultracentrifugation 100,000 × *g* at 10 °C for 90 minutes was carried out, and the resulting vesicle pellet resuspended in 500 µL of PBS.

### Dynamic Ligth scattering (DLS)

DLS measurements were performed with Zetasizer Nano-S (Malvern, Herfordshire, UK) at a controlled temperature of 25 °C. Scattering at 90° gives rise to particle size distributions that are deconvoluted from the raw scattering intensity using an exosome refractive index of 1.39 in the Stokes-Einstein equation. Three measurements of 13 averages were taken.

## Electronic supplementary material

Supplementary information
