# Supplementary material for: PLA2R binds to the annexin A2-S100A10 complex in human podocytes
Source: Sci Rep. 2017 Jul 31;7:6876. doi: 10.1038/s41598-017-07028-8 (PMC5537237; doi:10.1038/s41598-017-07028-8)
Supplement: Supplementary file 1 — Supplementary information [file 41598_2017_7028_MOESM1_ESM.pdf]

Supplementary information

PLA_2R binds to the annexin A2-S100A10 complex in human podocytes

Maryline Fresquet[1,2], Thomas A Jowitt[1], Edward A McKenzie[4], Matthew D Ball[4], Michael Randles[1,3], Rachel Lennon[1,3,5], Paul E Brenchley[2,5]

Supplementary Fig.1 - Western blot analysis of fractionated cellular protein from wild type and over-expressing PLA_2R podocytes

Supplementary Fig.2 - Characterisation of Ms monoclonal anti-PLA_2R [12-6-5]

Supplementary Fig.3 - Western blot analysis of fractionated cellular protein from over-expressing PLA_2R podocytes

Supplementary Fig.4 - A2t binding to lipid bilayer in Ca^{2+}-dependent manner

Supplementary Fig.5 - Co-localisation of PLA_2R/S100A10 at the cell surface and in extracellular vesicles

Supplementary Table 1 - Proteins identified by mass spectrometry as potential binding partners of PLA_2R extracellular domains

Supplementary Fig.1 – Western blot analysis of fractionated cellular protein from wild type and over-expressing PLA_2R podocytes

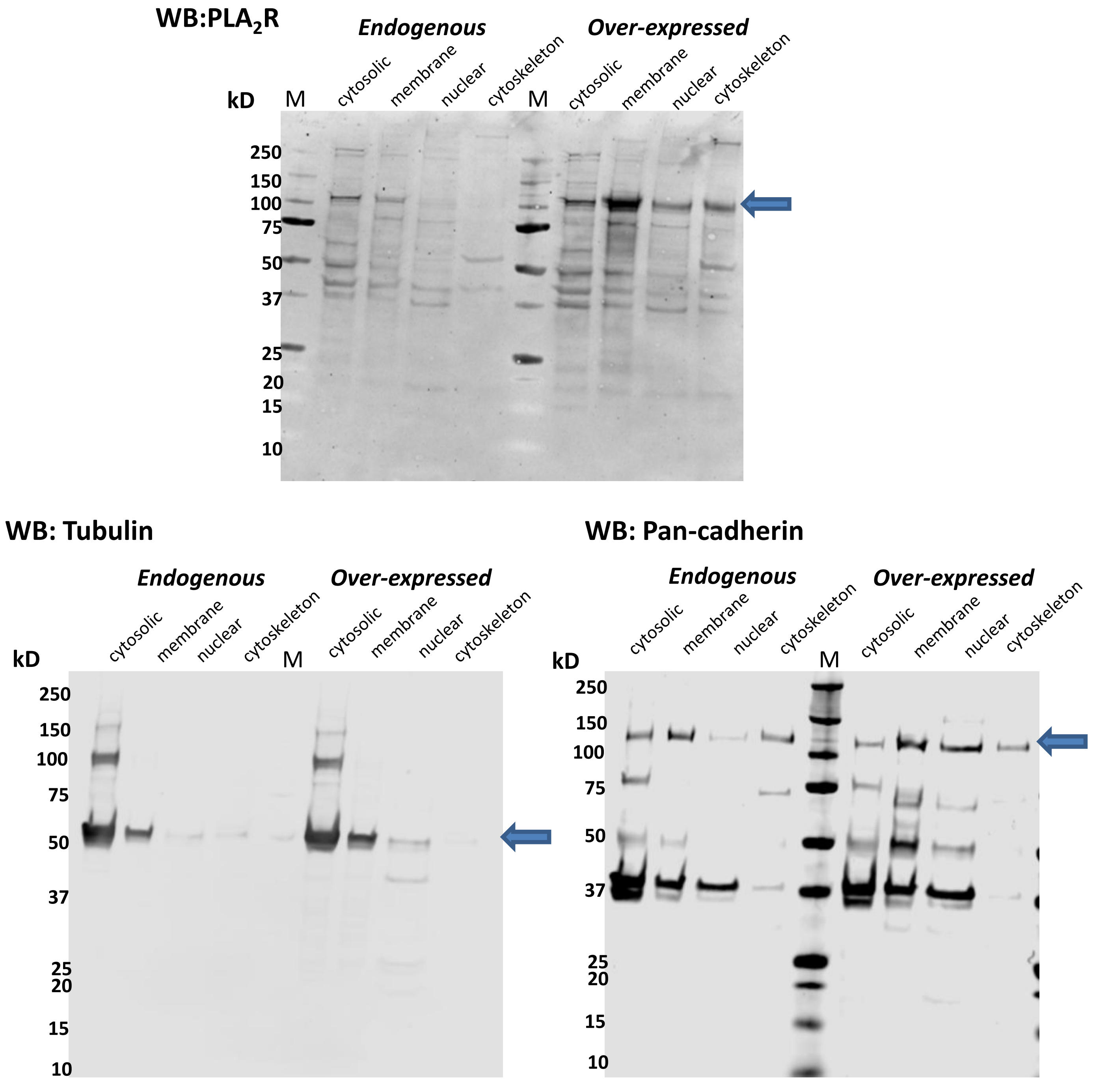

Western blot analysis of fractionated cellular protein from wild type and over-expressing PLA_2R podocytes

Podocytes proteins were fractionated using the subcellular protein extraction kit. Each extracts were analysed by western blotting using antibodies against PLA_2R, tubulin and cadherin (markers of cytoplasmic and membrane proteins, respectively). Arrows indicate the protein bands of interest for each blot. The results show an enrichment of PLA_2R protein in the membrane and membrane organelles fraction.

Supplementary Fig.2 - Characterisation of Ms monoclonal anti-PLA_2R [12-6-5]

a

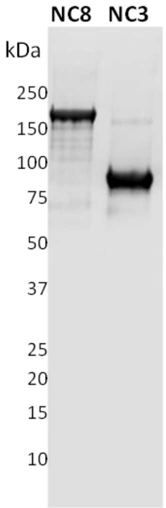

b

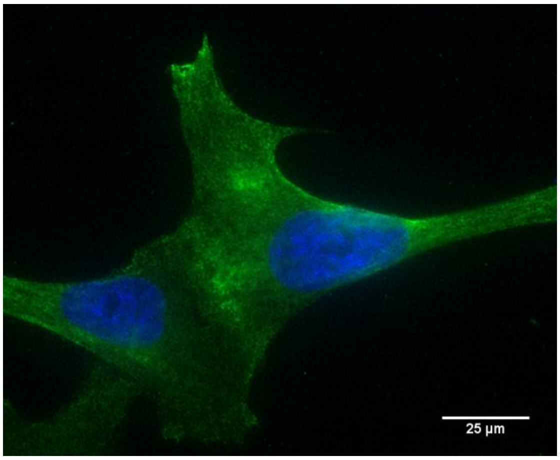

c

Kidney biopsy

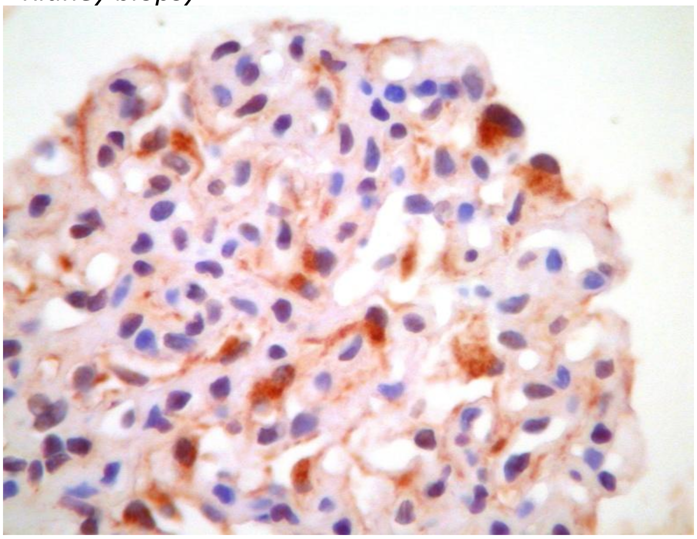

Anti-PLA_2R [12-6-5]

Source: Mouse. Antigen: PLA_2R N-terminus fragment (Alanine20- Proline663). Affinity: KD $5.4x10^{-10}$ M (determined by surface plasmon resonance between recombinant human PLA_2R and increasing concentration of Ms 12-6-5 antibody).

a) 1µg of recombinant full length extracellular domain of human PLA_2R (NC8) and a N-terminus fragment of PLA_2R (NC3) were subjected to SDS PAGE followed by **western blot** using Ms anti-PLA_2R (Cl12-6-5) at dilution 1:10000. b) **Immunofluorescence** image of PLA_2R over-expressing podocytes revealing cell surface expression of the PLA_2R receptor using Ms anti-PLA_2R (12-6-5) at dilution 1:400 (under 40x lens). c) **Immunohistochemistry** image of paraffin-embedded human kidney tissues stained with Ms anti-PLA_2R (12-6-5) at dilution 1:5000. Normal podocyte specific staining in an anti-PLA_2R negative MN kidney biopsy.

Supplementary Fig.3 – Western blot analysis of fractionated cellular protein from over-expressing PLA_2R podocytes

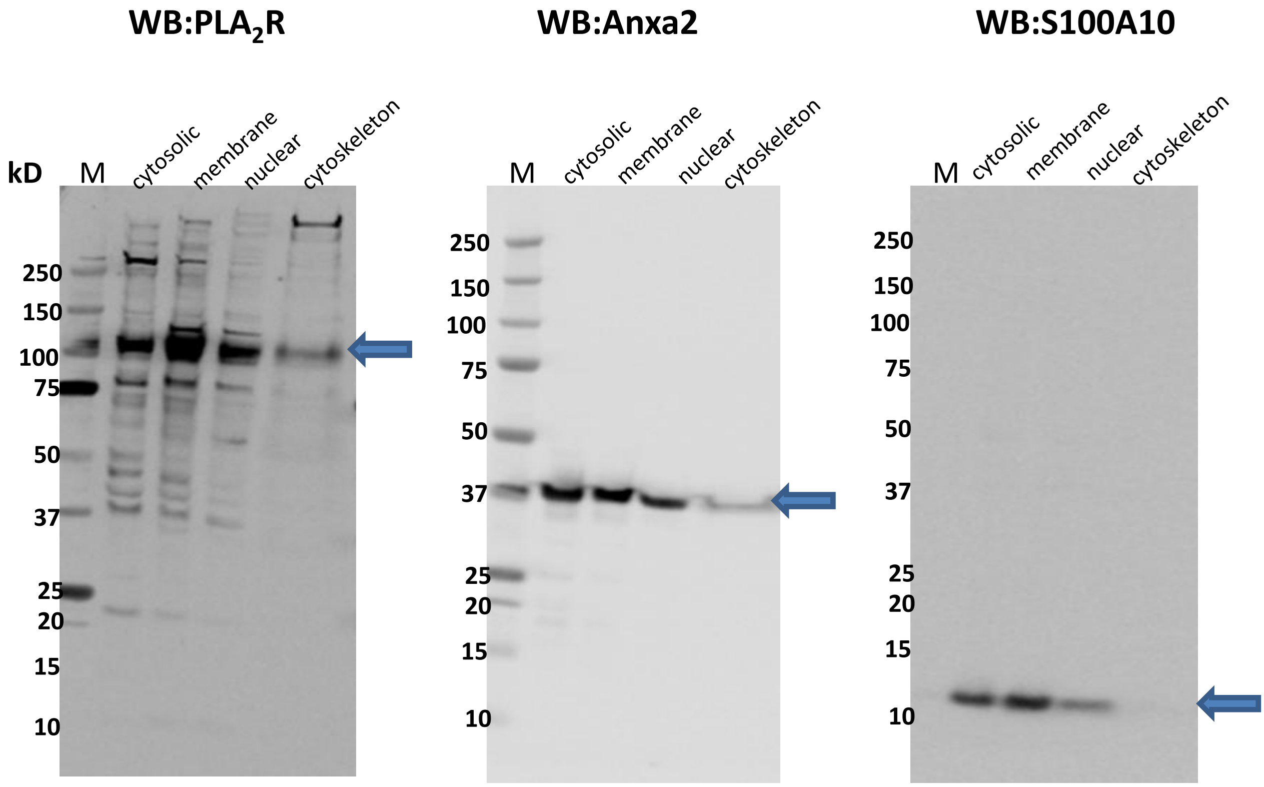

Western blot analysis of fractionated cellular protein from over-expressing PLA_2R podocytes

Podocytes proteins were fractionated using the subcellular protein extraction kit. Each extracts were analysed by western blotting using antibodies against PLA_2R, Anxa2 and S100A10. Arrows indicate the protein bands of interest for each blot. The results show an enrichment of all three blotted proteins in the membrane and membrane organelles fraction.

Supplementary Fig.4 - A2t binding to lipid bilayer in Ca^{2+}-dependent manner

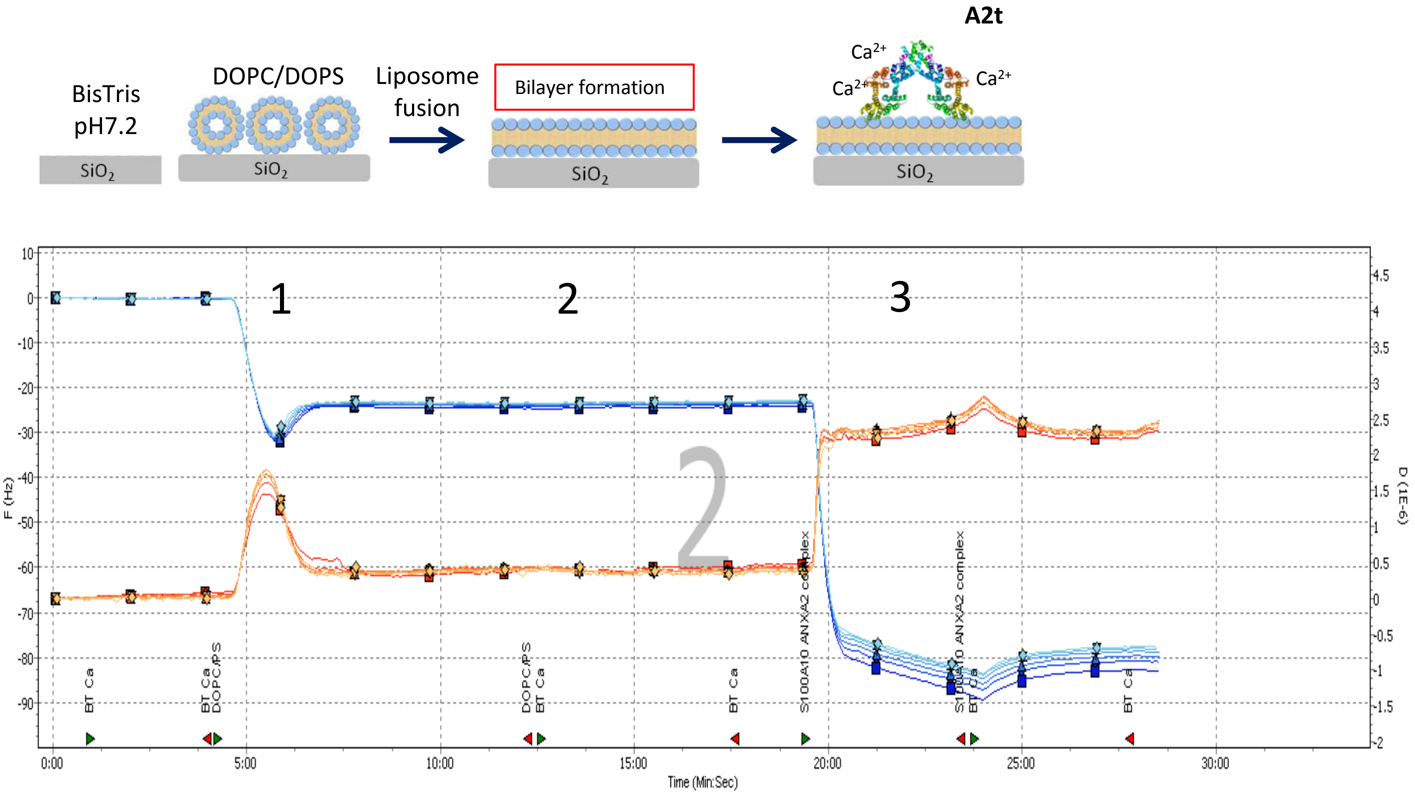

A2t binding to lipid bilayer in Ca^{2+}-dependent manner determined by Quartz Crystal Microbalance-D (Dissipation) or QCM-D

This QCM-D raw data demonstrates the formation of a DOPC/DOPS lipid bilayer followed by injection and binding of A2t complex in presence of Ca^{2+}. Frequency changes (Δf) are shown on the left axis and dissipation changes (ΔD) are shown on the right axis. Each lines represent different overtones (n=3, 5, 7, 9, 11 and 13). The injection of DOPC/DOPS lipids gives a large frequency and dissipation change indicating both mass adsorbed and increased viscoelasticity characteristics to the lipids deposition (1). The lipids rapidly fuse and form a stable, more rigid lipid bilayer (2). The rapid increase in mass (decrease in frequency) corresponds to the deposition of the A2t complex onto the lipids bilayer in presence of Ca^{2+} (3). Similar preformed lipid bilayer with anchored A2t complex was used in Figure 3B in order to determine the effects of pH with PLA_2R binding. Control experiments showed that PLA_2R NC8 and NC3 proteins did not bind to lipid bilayer alone (data not shown).

Supplementary Fig.5 - Co-localisation of PLA_2R/S100A10 at the cell surface and in extracellular vesicles

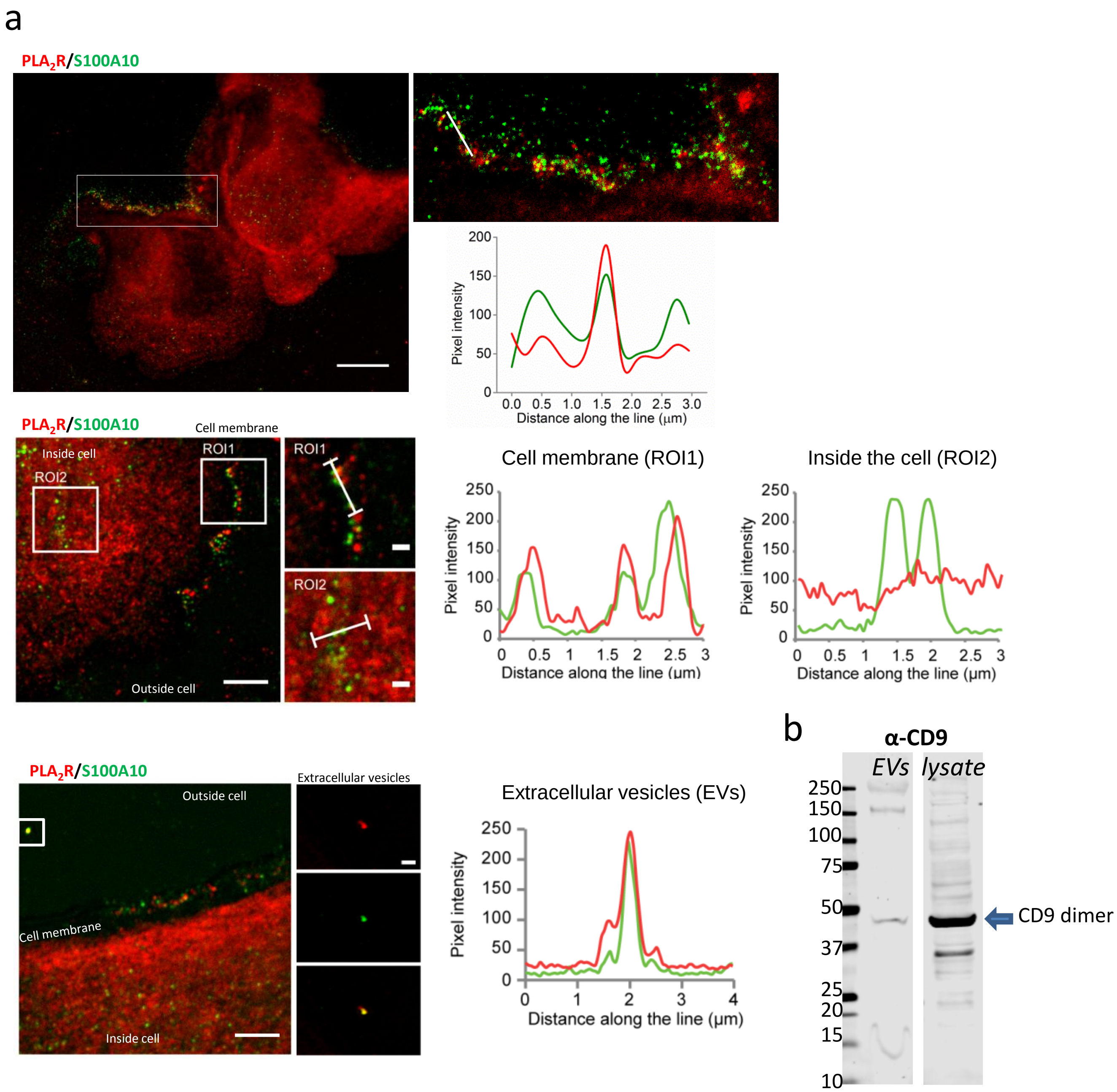

Co-localisation of PLA_2R/S100A10 at the cell surface and in extracellular vesicles

a) Co-localisation of PLA_2R/S100A10 at the cell surface and in vesicles. Podocytes cultured on coverslips were fixed and co-stained using antibodies against S100A10 (green) and PLA_2R (red) mAb 12-6-5. Merged images demonstrate regions of overlap (yellow) between the PLA_2R and S100A10 at the cell surface (top panels) and in vesicles (bottom panel) but not intracellularly. Scale bars, 10 μm (first image) and 5 μm (inset, 1 μm). Graph shows the intensity profile illustrating areas of co-localisation between the two proteins. b) Vesicles characterisation. Isolated EVs were analysed by western blotting using anti-CD9 antibody (rabbit polyclonal, Generon Ltd), a known marker of vesicles. A ~50Kda band was detected in EVs corresponding to the CD9 protein dimer and in the cell lysate (used as control).

Supplementary Table 1- Proteins identified by mass spectrometry as potential binding partners of PLA_2R extracellular domains

Protein ID	Accession #	MW	Number of peptides*		
			Pull down NC8 coated beads	QCM-D NC8	QCM-D lipid bilayer/NC8
PLA2R1 protein GN=PLA2R1	B7ZML4	168 kDa	168	56	20
**Annexin A2 GN=ANXA2**	**ANXA2**	**39 kDa**	**39**	**6**	**6**
Annexin A6 GN=ANXA6	ANXA6	76 kDa	15	0	0
Annexin A5 GN=ANXA5	ANXA5	36 kDa	12	0	2
Annexin (Fragment) GN=ANXA4	Q6LES2	36 kDa	8	0	0
Annexin A7 GN=ANXA7	ANXA7	53 kDa	6	0	0
Annexin A1 GN=ANXA1	ANXA1	39 kDa	4	0	2
Annexin A3 GN=ANXA3	ANXA3	36 kDa	3	0	0
Transforming growth factor-beta-induced protein ig-h3 GN=TGFBI	BGH3	75 kDa	17	3	0
Latent-transforming growth factor beta-binding protein 2 GN=LTBP2	G3V3X5	190 kDa	2	0	0
Versican core protein GN=VCAN	CSPG2	373 kDa	16	0	0
Cathepsin D GN=CTSD	CATD	45 kDa	8	2	0
Cathepsin Z GN=CTSZ	CATZ	34 kDa	4	0	0
Serpin H1 GN=SERPINH1	SERPH	46 kDa	11	0	0
SERPINB12 protein GN=SERPINB12	Q3SYB4	48 kDa	0	4	0
P4HA2 protein GN=P4HA2	Q05DA4	57 kDa	8	0	0
P4HA1 protein GN=P4HA1	P4HA1	61 kDa	4	0	0
Fibrillin-1 GN=FBN1	FBN1	312 kDa	5	0	2
Hornerin GN=HRNR	HORN	282 kDa	0	3	4
Laminin subunit beta-1 GN=LAMB1	G3XAI2	200 kDa	5	0	0
Protein-glutamine glutamyltransferase 2 GN=TGM2	B4DIT7	69 kDa	6	0	0
Filaggrin-2 GN=FLG2	FILA2	248 kDa	0	3	0
Nidogen-1 GN=NID1	NID1	136 kDa	3	3	0
Papilin GN=PAPLN	PPN	138 kDa	0	4	0
Hyaluronan and proteoglycan link protein 1 GN=HAPLN1	HPLN1	40 kDa	4	0	0
Protein-glutamine glutamyltransferase E GN=TGM3	TGM3	77 kDa	0	5	0
Insulin-like growth factor-binding protein 7 GN=IGFBP7	IBP7	29 kDa	0	4	0
Procollagen oxoglutarate dioxygenase 3 GN=PLOD3	PLOD3	85 kDa	5	0	0
Platelet-activating factor acetylhydrolase IB subunit gamma GN=PAFAH1B3	PA1B3	26 kDa	2	3	0
Cystatin-B GN=CSTB	CYTB	11 kDa	2	0	0
Inter-alpha (Globulin) inhibitor H2 GN=ITIH2	A2RTY6	106 kDa	2	0	0

*Spectral count – only proteins with greater than 95% probably and identified with 2-3 unique peptides were considered.
See supplemental method for experimental set up and samples preparation.
